# A Preliminary Study of Contrast-Enhanced Ultrasound (CEUS) and Cytokine Expression Analysis (CEA) as Early Predictors for the Outcome of Tibial Non-Union Therapy

**DOI:** 10.3390/diagnostics8030055

**Published:** 2018-08-24

**Authors:** Patrick Haubruck, Raban Heller, Michael C. Tanner, Volker Daniel, Gerhard Schmidmaier, Farhoud Bolourchi, Arash Moghaddam, Christian Fischer

**Affiliations:** 1 HTRG—Heidelberg Trauma Research Group, Center for Orthopedics, Trauma Surgery and Spinal Cord Injury, Trauma and Reconstructive Surgery, Heidelberg University Hospital, Schlierbacher Landstrasse 200a, 69118 Heidelberg, Germany; raban.heller@outlook.com (R.H.); michael.tanner@med.uni-heidelberg.de (M.C.T.); gerhard.schmidmaier@med.uni-heidelberg.de (G.S.); fbolourchi@web.de (F.B.); arash.moghaddam@klinikum-ab-alz.de (A.M.); christian.fischer@med.uni-heidelberg.de (C.F.); 2 Raymond Purves Bone and Joint Research Laboratories, Kolling Institute of Medical Research, Institute of Bone and Joint Research, University of Sydney, St. Leonards, New South Wales 2065, Australia; 3 Department of Transplantation Immunology, Institute of Immunology, University of Heidelberg, Im Neuenheimer Feld 305, 69120 Heidelberg, Germany; volker.daniel@med.uni-heidelberg.de; 4 ATORG—Aschaffenburg Trauma and Orthopedic Research Group, Center for Trauma Surgery, Orthopedics and Sports Medicine, Am Hasenkopf 1, 63739 Aschaffenburg, Germany

**Keywords:** bone regeneration, contrast-enhanced ultrasound, non-union, revision surgery, cytokine, diagnostics, prediction

## Abstract

The current study investigates if contrast-enhanced ultrasound (CEUS) or cytokine expression analysis (CEA) evaluating vascularization are capable of predicting the outcome of non-union therapy. Patients with tibial non-unions were surgically treated and participated in our follow-up program including perioperative collection of blood as well as CEUS analysis. Two groups were formed: Responders in group 1 (G1, *N* = 8) and Non-Responders in group 2 (G2, *N* = 5). Serum cytokine expression and local microperfusion were compared and correlated to the radiologic outcome. Evaluation of TNF-α expression revealed significantly lower values prior to first surgery in G1 (G1: 9.66 ± 0.96 pg/mL versus G2: 12.63 ± 1.2 pg/mL; *p* = 0.045); whereas after treatment both CEA and CEUS indicated a higher potential for angiogenesis in Responders. Logistic regression modelling revealed the highest predictive power regarding eventual osseous consolidation for the combination of both CEUS and serum CEA. The results provide first evidence regarding a link between changes in the serum expression of distinct pro-angiogenic cytokines and alterations in the local microperfusion assessed via both non-invasive and radiation-free diagnostic modalities. In addition, a combination of CEUS and CEA is a promising novel tool in early prediction of the outcome of non-union therapy.

## 1. Introduction

Fracture non-unions are common and occur in up to 30% [1]. It has been hypothesized that 100,000 fractures develop a non-union each year in the United States alone [2]. The tibia is most susceptible to failed bone regeneration [3] and resulting non-unions have severe implications for the patient’s quality of life [3]. The “diamond concept” defines core factors that are mandatory for successful bone regeneration and non-union healing, emphasizing the importance of sufficient vascularity [4,5,6]. In particular, the Masquelet-therapy was developed to treat large-defect-sized non-unions by inducing angiogenesis and enhancing local bone biology resulting ultimately in osseous regeneration [7]. Non-Responders to this therapy require additional extensive surgery ranging from repetition of the Masquelet-therapy [7] in early stages to distraction osteogenesis using Ilizarov fixators and eventual amputation of the affected limb [1,8]. Therefore, early identification of patients that are prone to high risk for treatment failure is necessary. Earliest radiologic determination (X-ray and computed tomography) regarding the success of treatment is only possible after 6 months [1,2] and is limited due to radiation exposure [2]. Therefore, radiation-free diagnostic alternatives capable of an early determination of the outcome are warranted.

Local and systemic analysis of the microperfusion, as sufficient vascularity is one of the pillars of bone regeneration [4], provides a novel and promising diagnostic approach [9,10,11]. The contrast-enhanced ultrasound (CEUS) is able to visualize the local tissue microperfusion on a capillary level. It is easily accessible, cost-efficient, and quick to use [9]. Furthermore, the used ultrasound contrast agent SonoVue^©^ (Bracco Imaging, Milan, Italy) has a low complication rate [10,12] and CEUS offers real-time examination in the contrast dynamics of the inspected tissues [9]. In a recent study, CEUS has been shown to be effective in monitoring the vascularity of non-unions [9] and distinguishing preoperatively based on the microperfusion between infected and non-infected non-unions [9]. In addition, bone regeneration correlates with changes in the serum expression pattern of pro-angiogenic and pro-inflammatory cytokines [2,13,14,15]. Recently, the systemic analysis of markers associated with bone regeneration assessed by measurement of serum cytokine expression analysis (CEA) was established as a valid method in the timely evaluation of biological processes occurring during fracture healing and bone regeneration [2,13,14,15]. Promising initial findings [2,9,13,16] support the predictive potential of both local (CEUS) and systemic (serum cytokine analysis) diagnostic modalities. However, no other study exists investigating the combined diagnostic potential of these novel radiation-free tools. Thus, this study was intended as a preliminary study providing first evidence regarding the predictive value of their combined use.

Hence, this study aims to determine primarily if local or systemic diagnostics alone or in combination are capable of predicting the outcome of non-union therapy. Secondary, the possibility of a potential link between changes in pro-angiogenic cytokines and local microperfusion is investigated. Ultimately, the possibility to determine a prognostic model regarding the outcome of non-union therapy based on a combination of parameters derived from both methods is evaluated. In particular, the current study aims to identify diagnostic models reflecting a higher potential of bone regeneration and tissue remodeling at an early stage during the applied non-union therapy.

## 2. Materials and Methods

### 2.1. Study Design and Patient Selection Process

**Figure 1:**
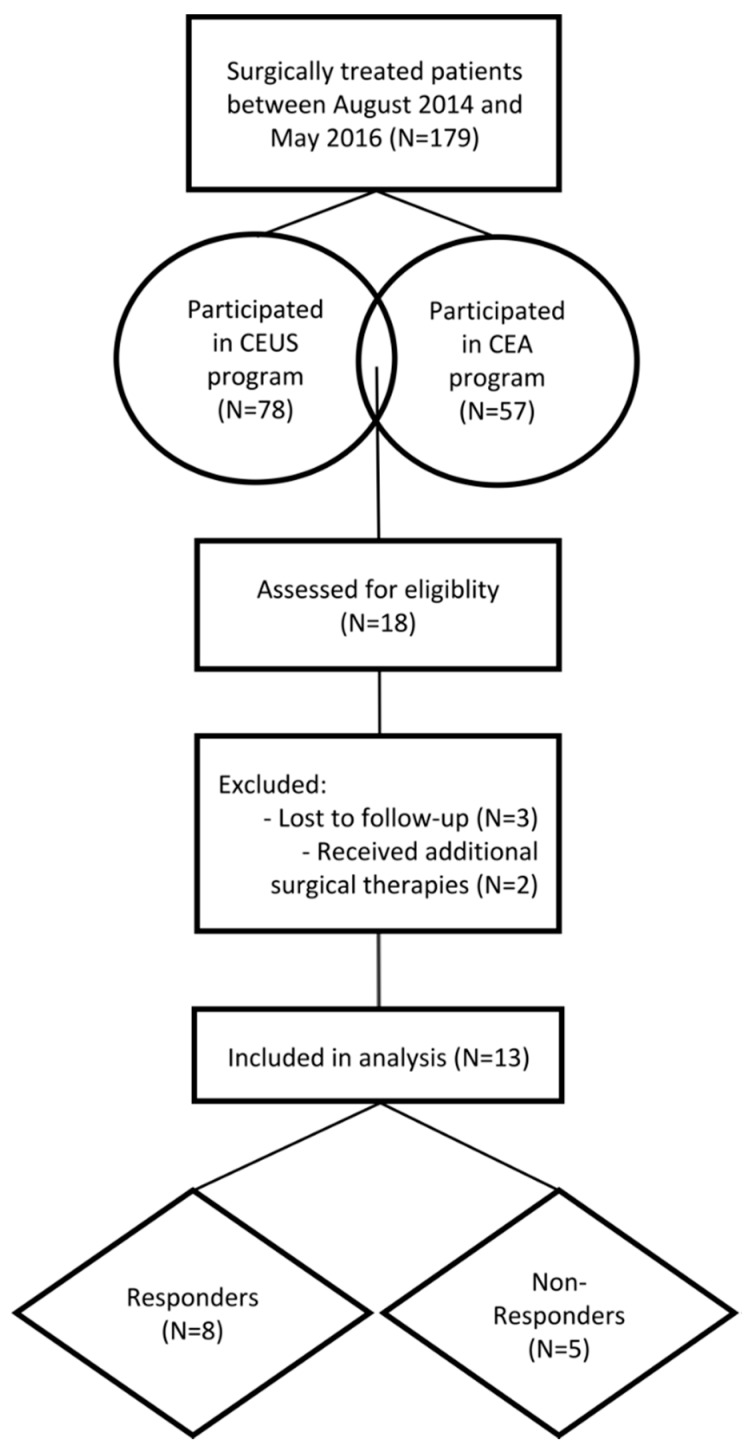
Flow diagram of the patient selection and exclusion process. CEUS, contrast-enhanced ultrasound; CEA, cytokine expression analysis.

This study was designed as a prospective clinical observer study. Between August 2014 and May 2016, 179 patients received surgical treatment of non-unions based on the “diamond concept” in our department. Out of these 179 patients, 78 patients participated in the perioperative CEUS program and 57 patients participated in the peripheral CEA program. A total of 18 patients agreed to participate in both programs. According to the inclusion and exclusion criteria, 13 (Responders in group 1 (G1), *N* = 8 and Non-Responders in group 2 (G2), *N* = 5) patients were included in the current study. The study was conducted in accordance with the declaration of Helsinki in its current form. Furthermore, inclusion of patients started subsequent to the approval by the local ethical committee (S-636/2011 (day of approval 16 July 2012) and S-033/2014 (day of approval 4 July 2014) (Figure 1).

### 2.2. Inclusion and Exclusion Criteria

Inclusion criteria were failed bone healing after diaphyseal fractures of the tibia, participation in both programs, age above 18, and a written declaration of consent. Exclusion criteria were the inability to provide written consent, chronic diseases, immunosuppressive medication, renal failure, and hepatic insufficiency. Patients who underwent revision surgery or additional surgical therapy during the study period were excluded from our study.

The inclusion and exclusion criteria were chosen to reduce possible confounders and to increase the consistency and thereby quality and reliability of our data and have been established in multiple previous studies [11,13,15].

### 2.3. Intervention

Depending on the type of non-union, a one- or two-step procedure was applied. One-step procedures included debridement of the non-union, de novo osteosynthesis, transplantation of autologous spongiosa, and additional application of bone morphogenetic proteins (BMPs). Two-step procedures were based on the Masquelet-therapy [17]. In the first step, radical debridement of the non-union was conducted and the resulting osseous defect filled with polymethyl methacrylate (PMMA) that induces the vascularized Masquelet membrane [18]. In a second step, the osseous defect is filled with autologous spongiosa and adjunct BMPs in order to provide a beneficial local microenvironment that results in bone regeneration by an osteoinductive, osteoconductive, and osteogenic stimulus [18]. De novo osteosynthesis utilizing plates, nails, or external fixators was performed during the first or the second step based on the stability of the affected long-bone. Intraoperatively, tissue samples were harvested and microbiologically analyzed for bacterial infection.

### 2.4. Analysis of the Cytokine Expression Pattern

Subsequent to the operative treatment, all patients underwent a standardized and established follow-up program that consisted of both clinical and radiologic examination as well as collection of blood samples [13]. Blood samples were collected preoperatively and additionally at days 2 and 7 after each step of the non-union treatment. Clinical and radiologic follow-up was scheduled 2 days postoperatively and after 6 weeks as well as 3, 6, and 12 months. The follow-ups consisted of clinical examination, a questionnaire (SF-12), and X-ray of the affected long bone. Patients included in the study were completing most of all follow-up visits; however, due to occasional unavailability of single patients at the scheduled follow-up, we were not able to obtain isolated blood samples. Venous blood samples were drawn (S Monovette 7.5 mL, Sarstedt AG, Nümbrecht, Germany) according to a highly standardized protocol [13]. Samples were taken between 0800 and 1100 on an empty stomach from all patients to reduce intraday measurement biases. Subsequently, the samples were centrifuged (1000 RPM, 10 min, 21 °C) and the serum was aliquoted and stored at −80 °C. Analysis of all serum samples was performed once all patients had concluded the 12-month follow-up at our institution. The quantitative analysis was performed with Luminex Performance Human High Sensitivity Assays (Quantikine^®^, RD Systems, Minneapolis, MN, USA) according to the manufacturer’s instructions. The lab technician performing the Luminex assays was blinded to both patient data and clinical outcome.

### 2.5. Contrast-Enhanced Ultrasound Examinations and Analysis

**Figure 2:**
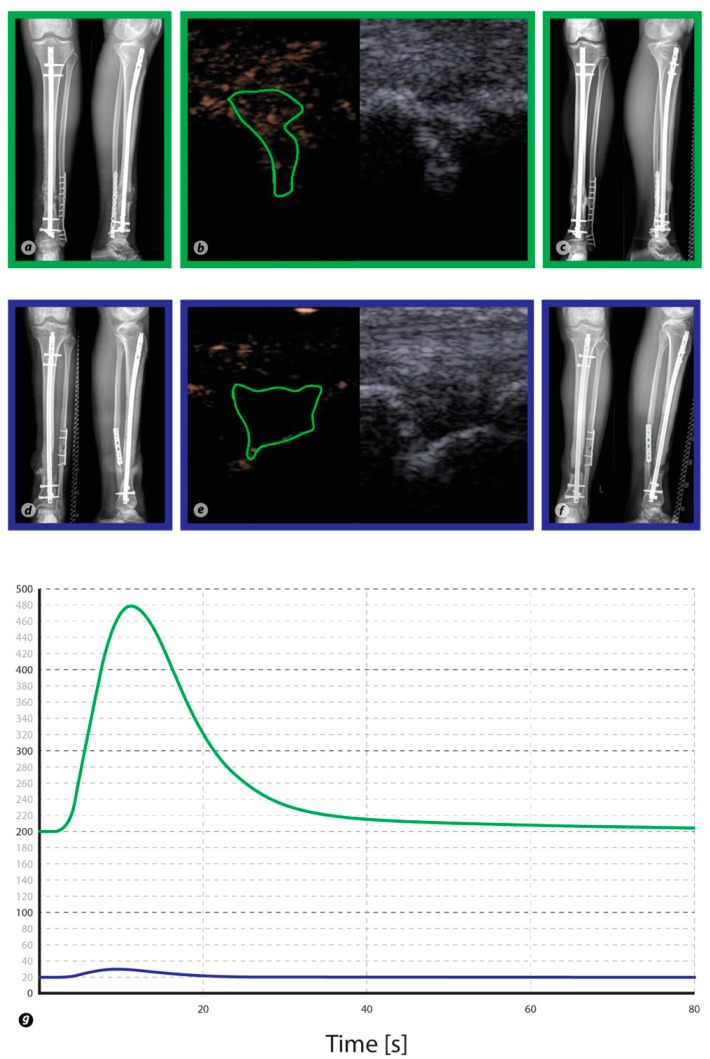
Conventional X-rays of the tibia and fibula as well as ultrasound images are shown for two patients. In particular, images in green rectangles derive from a Responder to the therapy, whereas images in blue rectangles derive from a Non-Responder. Panel (**a**,**d**) was taken prior to treatment, whereas (**c**,**f**) was taken 6 months after treatment and illustrates the respective outcome of therapy. Contrast-enhanced ultrasound images before (right side) and after contrast enhancement (left side) for each patient (**b**,**e**). The region of interest (irregular green shape) is placed into the non-union gap, and a time–intensity curve is generated by the VueBox quantification software (**g**). The time–intensity curve of the healing non-union exhibits stronger contrast enhancement than in persistent non-union.

CEUS was performed preoperatively and 3 months subsequent to the last surgical treatment of the non-union. CEUS examinations were based on a previously published protocol [9] and all ultrasound examinations were performed by the same experienced orthopaedic and trauma surgeon with a DEGUM (German Society for Ultrasound in Medicine) level III qualification, who was blinded towards the clinical outcome of the non-union treatment. First, utilizing the conventional B-mode (linear probe, 4–9 MHz, Acuson S3000 ultrasound device; Siemens Healthcare, Erlangen, Germany), the widest non-union gap was identified. Hereafter, a live dual-view B-mode image in the contrast mode was enabled and a 2.4-mL bolus of SonoVue^©^ (sulfur hexafluoride microbubbles with a phospholipide shell) was injected intravenously followed by 10 mL of a 0.9% saline solution. Immediately afterwards, a 2-min video clip with a frame rate of 5 Hz was digitally recorded. Settings complied with the recommendations of the European Federation of Societies for Ultrasound in Medicine and Biology (EFSUMB) [9,19]. The video clip was post processed with the dedicated CEUS quantification software VueBox^©^ (Bracco Imaging, Milan, Italy) allowing a semi-objective quantification of CEUS [9]. Time-intensity curves (TIC) were generated and perfusion parameters were calculated and compared between groups (Figure 2). In particular, the assessed parameters were [9]:

- Wash-in Rate (WiR in arbitrary units (a.u.), i.e., the maximum slope of the signal enhancement curve),
- Peak Enhancement (PE (a.u.), i.e., the maximum signal intensity of the enhancement curve),
- Rise Time (RT (s), i.e., the time a signal takes from baseline level to peak enhancement),
- Time to Peak (TTP (s), i.e., the duration from SonoVue^®^ application to Peak Enhancement).

### 2.6. Evaluation of Outcome

The clinical and radiologic outcome of the non-union therapy was evaluated at the 12-month follow-up consultation and patients were declared as Responders or Non-Responders based on clinical signs of mechanical stability and full weight bearing as well as radiologic signs of consolidation (sufficient bridging apparent in at least three of four cortices) [20,21,22]. Responders were then assigned to group 1 (G1) and Non-Responders to group 2 (G2).

### 2.7. Mathematical Modelling Process

Investigation of the predictive power of variables regarding consolidation was carried out via multivariate logistic regression modelling including both cytokine and CEUS data. All values were z-standardized before calculation. Due to the limited sample size, variables were combined to avoid an overfitting of the final model. In both CEUS and CEA, all variables characterized by a *p*-value < 0.10 as assessed in the test for group differences regarding consolidation were identified as variables of interest (VOI). For both groups, the combination was calculated by:

1. Estimation of the mean of each variables’ subgroup of non-consolidation and consolidation,
2. If the mean of the non-consolidation was higher than in the consolidation group, the variables values were added to those of the variable characterized by the lowest *p*-value, while if the non-consolidation was lower than in the consolidation group values were subtracted from those of the variables mentioned above.

The resulting pathway and final variables are given in the results section. Model selection was performed via AIC (Akaike Information Criterion) comparison [23] providing an estimate of the relative information loss compared to another given model. The AIC deals with the corresponding tradeoff between a given simplicity of a model and the goodness of fit and thus provides additional information regarding the quality of a model relative to another. The AUC (Area Under the Curve) of the ROC (Receiver Operating Characteristic) Curve and the respective confidence interval were used to assess the predictive performance. In the present study, the AUC estimates the chances of assigning a higher model-based score to patients who actually show consolidation as compared to those who do not.

### 2.8. Statistics

As an analogue to the design of our previous prospective explorative studies [16,24,25,26], correlation analyses were conducted between all variables. When normal distribution was not given as assessed in the Shapiro–Wilk Test, non-parametric test methods were assessed to investigate location shifts between (Mann–Whitney *U*-Test) as well as within subgroups at different time points (Wilcoxon Signed Rank Test). Categorical variables were evaluated using the Chi-square test. All *p*-values quoted are to be interpreted in a descriptive way as they were not adjusted for multiple testing as this is an exploratory post-hoc analysis comparable to our previous study designs. Differences were considered significant below the significance level of *p* = 0.05. All statistical calculations were performed with R version 3.4.4 [27] and the package “pROC” for receiver operator characteristics (ROC) analysis [28]. Figures were created by using the package “ggplot2” [29].

## 3. Results

### 3.1. Patient Demographics

#### 3.1.1. Responders (G1)

A total of four male and four female patients with an average age of 41.1 ± 16 years were included into G1 (*N* = 8). The average body mass index (BMI) was 29.5 and patients were previously treated with a mean of 2 surgeries. The time interval between initial fracture and non-union surgery was 38.4 months on average. However, one patient was treated 226 months after the initial fracture. One patient was an active smoker, four patients were non-smokers, and three patients were former smokers [30]. None of the patients showed clinical signs of a local infection; however, microbiological results showed that five patients had an infected non-union. The average NUSS (Non-union scoring system) score [31] was 44 (Table 1).

#### 3.1.2. Non-Responders (G2)

A total of four male and one female patients with an average age of 51.9 ± 9.8 years were included into G2 (*N* = 5). The average BMI was 29.5 and patients were previously treated with a mean of 3 surgeries. The time interval between initial fracture and non-union surgery was 84 months on average; however, one patient was treated 339 months after the initial fracture. One patient was an active smoker and four patients were non-smokers [30]. None of the patients showed clinical signs of local infection; however, microbiological results showed that all patients had an infected non-union. The average NUSS (Non-union scoring system) score [31] was 42.4 (Table 1).

### 3.2. Contrast-Enhanced Ultrasound

**Figure 3:**
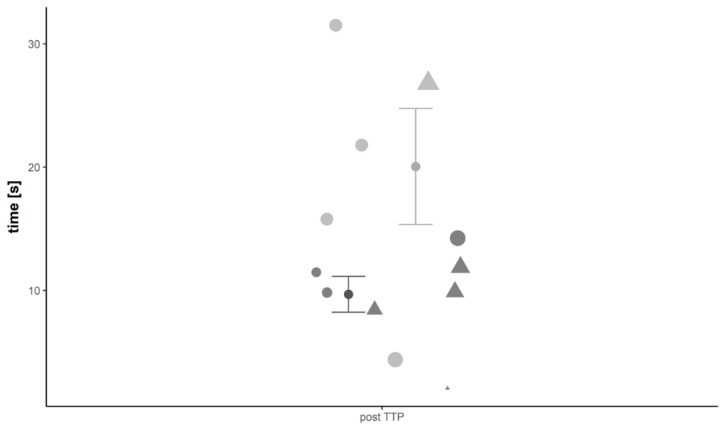
Visualization of results from CEUS analysis. In particular, results from the analysis of the parameter time to peak (TTP) 12 weeks subsequent to the second step are shown and compared between groups. Dark points with whiskers display mean time in Responders, grey points with whiskers indicate mean time in Non-Responders (whiskers are the standard error of the mean (SEM). Results are further stratified to age and sex and values for each participating patient are given. Here, triangle indicates male sex and dots female sex, size of symbol increases with age of patient.

Preoperative CEUS quantification of the included tibial non-unions revealed a higher WiR in patients not responding to the non-union treatment compared to patients that showed successful non-union consolidation (WiR: G1: 35.14 ± 11.32 a.u. versus G2: 50.43 ± 23.17 a.u., *p* > 0.05). In addition, Responders to the therapy had a slower contrast inflow with a mean RT of 16.38 ± 6.93 s versus 8.96 ± 1.69 s in G2 (*p* > 0.05). It took 26.8 ± 10.2 s on average in G1 for the contrast agent to reach the signal intensity peak (TTP in G2: 10.6 ± 1.7 s, *p* > 0.05) and the mean PE was lower than in G2 (PE in G1: 189.17 ± 57.16 a.u. versus G2: 218.63 ± 95.65 a.u., *p* > 0.05). The postoperative WiR was higher in Responders compared to Non-Responders (WiR in G1: 106.59 ± 34.45 a.u. versus G2: 46.91 ± 21.47 a.u., *p* > 0.05). Furthermore, postoperative contrast inflow was faster in G1 than in G2 (RT in G1: 5.1 ± 1 s versus G2: 15.5 ± 4.2 s, *p* > 0.05) and the signal intensity peak was higher (PE in G1: 318.82 ± 98.56 a.u. versus G2: 252.39 ± 92.11 a.u., *p* > 0.05) and reached in a shorter amount of time (TTP in G1: 9.7 ± 1.5 s versus G2: 20.1 ± 4.0 s, *p* > 0.05) (Figure 3) in Responders to the therapy (Table 2).

### 3.3. Serum Cytokine Expression Analysis

**Figure 4:**
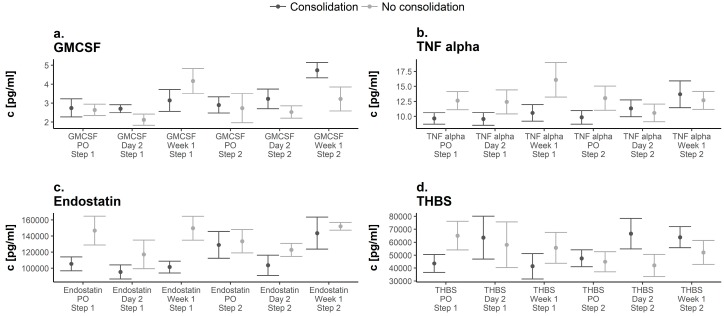
Expression pattern of GM-CSF (**a**), TNF-α (**b**), Endostatin (**c**), and Thrombospondin-2 (THBS) (**d**) over time in both groups. Dark points display Responders, grey points indicate Non-Responders. Significant differences are indicated by a star (*p* < 0.05, whiskers are SEM). Abbreviations: preoperatively = PO.

Evaluation of GM-CSF serum expression levels showed similar preoperative values in both groups (Prior to first surgery; G1: 2.75 ± 0.48 pg/mL versus G2: 2.64 ± 0.24 pg/mL, *p* > 0.05). Hereafter, expression levels increased in both groups until 1 week after the initial surgery (Figure 4a). G2 showed peak levels 1 week after the first surgery (4.17 ± 0.52 pg/mL). Serum expression levels prior to the first and second step were similar in both groups and levels increased after the second surgery. G1 showed peak expression levels 1 week after the second surgery (4.74 ± 0.41 pg/mL) (Figure 4a). Evaluation of TNF-α serum expression levels revealed significantly lower values preoperatively in G1 compared to G2 (prior to first surgery; G1: 9.66 ± 0.96 pg/mL versus. G2: 12.63 ± 1.2 pg/mL; *p* = 0.045). Then, expression levels in G1 remained lower compared to those in G2 until 2 days subsequent to the second step of the non-union therapy when serum levels of TNF-α were higher for the first time in Responders to the therapy and remained higher onward (1 week subsequent to the second surgery; G1: 13.69 ± 2.2 pg/mL versus G2: 12.67 ± 1.25 pg/mL, *p* = 0.056) (Figure 4b). Serum expression analysis of endostatin revealed lower values preoperatively in G1 (G1: 105,398.24 ± 8697.62 pg/mL versus G2: 146,633.94 ± 14,188.26 pg/mL, *p* > 0.05). Hereafter, serum expression levels in G2 were higher during the course of treatment. Peak values in both groups were reached 1 week subsequent to the second surgery and were slightly higher in G2 (G1: 143,499.28 ± 19,889.61 pg/mL versus G2: 151,945.74 ± 4171.04 pg/mL, *p* > 0.05) (Figure 4c). Analysis of thrombospondin-2 revealed lower values in G1 compared to G2 preoperatively to the first surgery (G1: 43,575.34 ± 6927.84 pg/mL versus G2: 65,048.9 ± 8784.59 pg/mL, *p* > 0.05). Subsequently, values in G2 decreased until 1 week after the second surgery and values remained below the initial serum expression levels during the course of treatment. In contrast, values in G1 showed peak values both 2 days after the first and second surgery (2 days subsequent to first surgery, G1: 63,583.72 ± 16,563.04 pg/mL; 2 days subsequent to second surgery, G1: 66,594.91 ± 11,820.5 pg/mL) (Figure 4d).

### 3.4. Variable Selection and Computation

**Figure 5:**
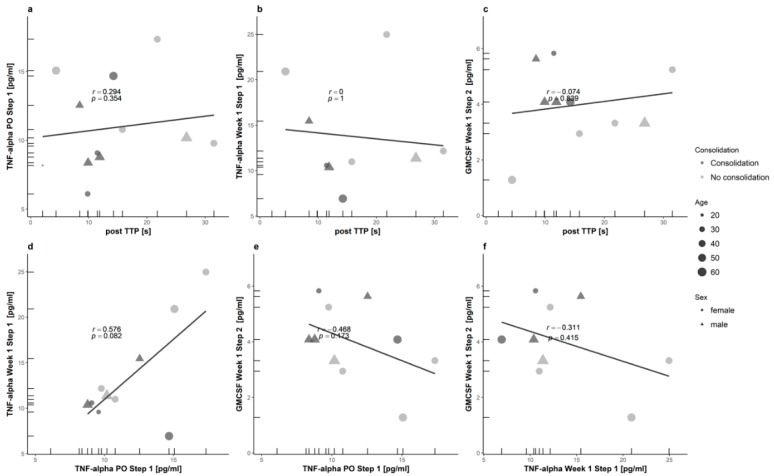
Correlation of the variables of interest (VOIs). Here, correlations between CEUS and CEA parameters are depicted in panel (**a**–**c**) and correlations between CEA parameters in panel (**d**–**f**) (postTTP vs. TNF.α.PO.Step.1 (**a**), vs. TNF.α.Week.1.Step.1 (**b**) and vs. GM.CSF.Week.1.Step.2 (**c**)//TNF.α.PO.Step.1 vs. TNF.α.Week.1.Step.1 (**d**) and vs. GM.CSF.Week.1.Step.2 (**e**)//TNF.α.Week.1.Step.1 vs. GM.CSF.Week.1.Step.2 (**f**)). Covariates are displayed via size (age) and shape (sex) of each data point. The color indicates the criterion (presence or absence of consolidation).

Correlation of VOIs is shown in detail in Figure 5. According to the selection process as described in the methods section, the CEUS variable “post TTP” and the cytokines “GMCSF Week 1 Step 2”, “TNF-α PO Step 1” and “TNF-α Week 1 Step 1” were identified to form the variables subgroups coefficients. The computation process revealed the following term:

Variables of Interest (VOI):

(A) CEUS-01 = (postTTP)
(B) Cytokines-01 = (TNF.α.PO.Step.1)
(C) Cytokines-02 = (TNF.α.Week.1.Step.1)
(D) Cytokines-03 = (GM.CSF.Week.1.Step.2)

Formulas:

(1) (CEUS variable) = ((A))
(2) (Cytokines variable) = ((B)) + ((C)) – ((D))
(3) (Together) = ((1)) + ((2))

### 3.5. Analysis of the Prognostic Performance

**Figure 6:**
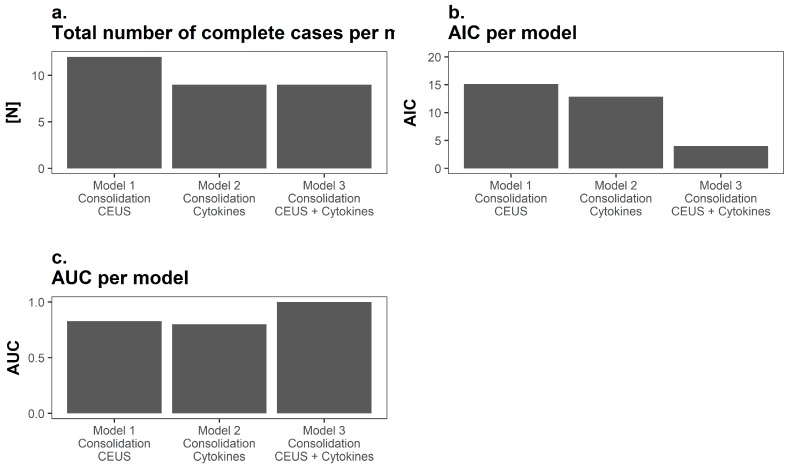
Visualization of the prognostic performance. (**a**) Only complete cases were included in the mathematical modelling process and due to single missing values analyzed cases were fewer once CEA was included; (**b**) Here the Akaike Information Criterion (AIC) is depicted for each respective model. Interestingly, despite fewer cases, the AIC was lowest in the mathematical model including parameters from both modalities (low AIC indicating better prognostic performance); (**c**) Similarly, the area under curve (AUC) was higher in the mathematical model including parameters from both modalities.

Logistic regression modelling revealed the highest predictive power regarding consolidation subsequent to the non-union treatment for parameters generated by using both CEUS (TTP 3 months after surgery) and serum CEA (GM-CSF values 1 week after the second surgery, TNF-α values both prior to the initial surgery and 1 week after the initial surgery). Thus, model selection was performed using the models AIC and AUC of the corresponding ROC analysis. Only complete cases without a single missing value were integrated in the prediction modelling process; therefore, the number of complete cases was lower once serum cytokine parameters were included due to single missing values (Figure 6a). It is noteworthy that, despite the number of completed cases being lower in the models including serum cytokine parameters, AUC was the highest and AIC the lowest for models including both CEUS and cytokine parameters (Figure 6b,c).

## 4. Discussion

This study aimed to investigate the diagnostic performance of CEUS and peripheral CEA regarding the outcome of non-union therapy based on the “diamond concept”. The results indicate that predictive models including parameters from both CEUS and CEA together perform better compared to models including parameters solemnly from a single modality.

Initial studies investigating in the diagnostic performance of CEUS in context with tibial non-unions provided promising first results regarding the capabilities of CEUS. A recent study by Fischer et al. [9] showed that a hypervascularized non-union gap correlates significantly with the microbial testing regarding an infected non-union during qualitative assessment [9]. Furthermore, CEUS quantification of infected non-unions revealed a higher WiR, a faster contrast inflow, and a higher PE compared to aseptic non-unions [9]. Fischer et al. concluded that CEUS may be used in real-time to monitor the vascularity of non-unions [9]. In a subsequent study, CEUS analysis was introduced as a promising modality in predicting eventual osseous consolidation based on perfusion [32]. Another study by Xu et al. [33] used CEUS for the assessment of vascularization of hydroxyapatite orbital implants. The results indicated that CEUS is useful for the assessment of vascularization of hydroxyapatite orbital implants and provides better visualization of the dynamic process than contrast-enhanced magnetic resonance imaging [33]. In the current study, CEUS analysis indicated a hypervascularization of non-unions on a capillary level in Non-Responders to the therapy prior to treatment. Intraoperative microbial testing revealed a high percentage of clinically occult infections in both groups with absent signs of infection in any of the patients except a hypervascularization detected by CEUS. These findings are supported by a previous study establishing CEUS as a highly sensitive diagnostic modality in revealing occult infections [11]. In these particular infections, assessment of serological parameters, such as c-reactive protein and leukocyte count, as well as clinical parameters are known to be insufficient [11]. Non-union therapy based on the “diamond concept” is an effective treatment of infected non-unions [6] by utilizing radical debridement of infected tissue. Three months postoperatively, Responders to the non-union therapy showed a higher vascularization compared to patients that did not respond. This might be linked to an improved vascularization of the graft and therefore a higher possibility of consolidation of the non-union. The results of the current study indicate that an initial high vascularization in patients with a persistent non-union might be due to a persistent active inflammation disadvantageous for the outcome of non-union treatment. In contrast, elevated vascularization of non-unions 3 months after the treatment might be due to an improved vascularization of the graft and therefore indicate a favorable local microenvironment regarding the outcome of therapy [32].

Endostatin is a well-known endogenous inhibitor of angiogenesis [34] by its binding to integrin alpha5 beta-1, which is also expressed on osteoblasts, osteoclasts, and chondrocytes [34]. A recent study by Holstein et al. investigated if endostatin inhibits callus remodelling during fracture healing and concluded that endostatin might be involved in bone healing disturbances [34]. In the current study, serum levels of endostatin were constantly higher in patients that did not respond to the non-union treatment during the course of the study. Higher levels of endostatin might correlate with an impaired vascularization of the graft and therefore a disadvantageous microenvironment for osseous consolidation. These findings are supported by the results of the CEUS analysis showing a decreased vascularization subsequent to grafting of the osseous defect in patients not responding to the therapy.

Thrombospondin-2 is highly expressed in the early mesenchymal phase of fracture healing [35], and recent studies indicated that thrombospondin-2 plays an important role in the regulation of early fracture mesenchyme [36]. In addition, the presence of thrombospondin-2 may influence the course of mesenchymal callus progenitors away from chondrogenic pathways and toward osteogenesis [35]. In the current study, thrombospondin-2 was elevated early after each surgical treatment in Responders to the therapy. Multiple factors must interoperate to maintain early callus mesenchyme [36]. Therefore, an early higher expression of thrombospondin-2 might correlate with a tendency of the bone to maintain regular fracture healing.

Granulocyte macrophage colony stimulating factor (GM-CSF) initiates the sprouting phase of angiogenesis and promotes the stabilization of new microvessels [37]. However, the role of GM-CSF in bone healing is unknown [37]. A recent study by Yan et al. investigated the role of GM-CSF during wound healing. Yan et al. concluded that GM-CSF accelerates wound healing by augmenting the microvascular barrier integrity during wound healing [37]. Interestingly, in the current study GM-CSF levels in Responders were the highest 1 week after the second surgery whereas levels in Non-Responders were the highest subsequent to the first surgery. A higher expression of GM-CSF after the second surgery might correlate with an enhanced microvascularity and angiogenesis during the initial integration of the graft contributing towards a higher rate of consolidation. This finding is also supported by the higher microperfusion detected via CEUS analysis. Hence, results of the current study introduce a link between elevated GM-CSF levels during integration of the graft and an increased local microperfusion.

TNF-α has been shown to promote angiogenesis [13]. In addition, TNF-α is able to trigger apoptosis or secure the survival of cells [38]. In the current study, expression of TNF-α was lower in Responders to the therapy compared to Non-Responders until 2 days after the second surgery. Then, TNF-α levels were higher in Responders. This cytokine switch might correlate with an enhanced angiogenesis and augmented activation of TNFR-2 resulting in a stimulus for bone regeneration [9] during the early integration of the graft. Enhanced angiogenesis caused by this cytokine switch is supported by the findings of the CEUS analysis visualizing an improved vascularization after the second surgery.

### Limitation

The results are limited due to inevitable limitations. Despite the severity of non-unions, they remain an infrequent complication. Thus, the potential number of eligible patients is small from the beginning. In addition, only a small group of patients agreed to participate in both programs. This resulted in a considerably reduced number of eligible participants for the current study. Ultimately, we were able to include only a small number of patients into the current study due to our strict inclusion and exclusion criteria. The findings are supported by data gathered from larger samples derived from both the CEA [2,13,15] and CEUS [9,32] program. In these studies, we utilized the same methods as in this study. Thereby, the likelihood of reporting correct results despite the small sample size is supported by this previous data and increases the validity of our data. In addition, the utilization of both diagnostic modalities is highly standardized in our hospital resulting in higher consistency and minimized bias and variability. Rigorous and conservative statistical testing was performed to analyze data and account for the small sample size. Both the standardized approach and the thorough analysis were intended to increase the credibility of our data and make the findings more robust. Nevertheless, due to the limited number of patients the results of the current study have to be interpreted carefully and studies with higher patient numbers are necessary to validate the findings of the current study. Measurements of serum expression of cytokines are highly sensitive and might be influenced by a variety of factors. However, the validity of serum expression analysis using Luminex assays has been proven by multiple studies [2,13]. Therefore, we believe that this does not implicate the findings of this study. Ultrasound examinations are limited to the subjectivity of the examiner. However, due to the standardized approach, the single experienced examiner, and the semi-objective evaluation using a quantification software we believe possible confounders were minimized.

## 5. Conclusions

The results of the current study provide first evidence regarding a link between changes in the serum expression of distinct angiogenic cytokines and alterations in the local microperfusion assessed via both non-invasive and radiation-free diagnostic modalities. In addition, the results from both CEUS and CEA indicate a higher potential for angiogenesis after the second step of the non-union treatment in patients that show consolidation. Multiple studies have shown a link between angiogenesis and fracture healing [36]; improving and maintaining angiogenesis remains one of the key factors in the “diamond concept” [5]. Therefore, both CEA and CEUS might be valid instruments in evaluating the outcome of non-union therapy. However, binary logistic regression modelling revealed a higher predictive performance regarding consolidation once both modalities were combined. Therefore, the results of the current study indicate that a combination of CEUS and peripheral CEA is a promising novel tool in early prediction of the outcome of non-union therapy.

## Figures and Tables

**Table 1:** Patient characteristics.

Patients		All	Responders (G1)	Non-Responders (G2)
Sex	Male	8	4	4
	Female	5	4	1
Age		45.3 ± 14.9	41.1 ± 16	51.9 ± 9.8
BMI		29.5 ± 7.8	29.5 ± 9.3	29.5 ± 3.2
Infection	Yes	10 (77%)	5 (62.5%)	5 (100%)
	No	3 (23%)	3(27.5%)	0 (0%)
Smoking	S	2	1	1
	NS	8	4	4
	FS	3	3	0
Trauma to Non-Union Therapy (Months)		55.9 ± 99.4	38.38 ± 71	84 ± 127.9
NUSS Score		43.38 ± 6	44 ± 6.2	42.4 ± 5.7
Localisation	Tibia	13	8	5
Fixation	Nail	12	7	5
	Plate	1	1	0
Previous surgeries		2.36 ± 1.1	2.0 ± 0.5	3.0 ± 1.41

Abbreviations: Active smoker (S); Non-smoker (NS); Former smoker (FS); Body mass index (BMI); Non-union scoring system (NUSS). Age is presented in years.

**Table 2:** CEUS parameters.

Parameter	Time Point	Patients	
		Responder (G1; *N* = 8)	Non-Responder (G2; *N* = 5)
**WiR (a.u.)**	Preoperative	35.14 ± 11.32	50.43 ± 23.17
	3 months postoperative	106.59 ± 34.45	46.91 ± 21.47
**TTP (s)**	Preoperative	26.77 ± 10.15	10.56 ± 1.71
	3 months postoperative	9.68 ± 1.45	20.05 ± 3.98
**RT (s)**	Preoperative	16.37 ± 6.94	8.96 ± 1.69
	3 months postoperative	5.08 ± 1.00	15.50 ± 4.17
**PE (a.u.)**	Preoperative	189.12 ± 57.15	218.63 ± 95.65
	3 months postoperative	318.82 ± 98.56	252.39 ± 92.11

Abbreviations: Wash in rate (WiR); Time to peak (TTP); Rise time (RT); Peak enhancement (PE; seconds (s); arbitrary units (a.u.).

## References

[1] Schmidmaier G., Moghaddam A. (2015). Long bone nonunion. Zeitschrift fur Orthopadie und Unfallchirurgie.

[2] Moghaddam A., Breier L., Haubruck P., Bender D., Biglari B., Wentzensen A., Zimmermann G. (2016). Non-unions treated with bone morphogenic protein 7: Introducing the quantitative measurement of human serum cytokine levels as promising tool in evaluation of adjunct non-union therapy. J. Inflamm..

[3] Brinker M.R., Hanus B.D., Sen M., O’Connor D.P. (2013). The devastating effects of tibial nonunion on health-related quality of life. J. Bone Jt. Surg..

[4] Giannoudis P.V., Einhorn T.A., Marsh D. (2007). Fracture healing: The diamond concept. Injury.

[5] Calori G.M., Giannoudis P.V. (2011). Enhancement of fracture healing with the diamond concept: The role of the biological chamber. Injury.

[6] Moghaddam A., Zietzschmann S., Bruckner T., Schmidmaier G. (2015). Treatment of atrophic tibia non-unions according to ‘diamond concept’: Results of one- and two-step treatment. Injury.

[7] Masquelet A.C., Begue T. (2010). The concept of induced membrane for reconstruction of long bone defects. Orthoped. Clin. N. Am..

[8] Yin P., Ji Q., Li T., Li J., Li Z., Liu J., Wang G., Wang S., Zhang L., Mao Z. (2015). A systematic review and meta-analysis of ilizarov methods in the treatment of infected nonunion of tibia and femur. PLoS ONE.

[9] Fischer C., Nissen M., Schmidmaier G., Bruckner T., Kauczor H.U., Weber M.A. (2017). Dynamic contrast-enhanced magnetic resonance imaging (dce-mri) for the prediction of non-union consolidation. Injury.

[10] Piscaglia F., Bolondi L., Italian Society for Ultrasound in Medicine and Biology Study Group on Ultrasound Contrast Agents (2006). The safety of sonovue in abdominal applications: Retrospective analysis of 23,188 investigations. Ultrasound Med. Biol..

[11] Fischer C., Preuss E.M., Tanner M., Bruckner T., Krix M., Amarteifio E., Miska M., Moghaddam-Alvandi A., Schmidmaier G., Weber M.A. (2016). Dynamic contrast-enhanced sonography and dynamic contrast-enhanced magnetic resonance imaging for preoperative diagnosis of infected nonunions. J. Ultrasound Med..

[12] Jaschke M., Weber M.A., Fischer C. (2018). CEUS-application possibilities in the musculoskeletal system. Der Radiologe.

[13] Haubruck P., Kammerer A., Korff S., Apitz P., Xiao K., Buchler A., Biglari B., Zimmermann G., Daniel V., Schmidmaier G. (2016). The treatment of nonunions with application of bmp-7 increases the expression pattern for angiogenic and inflammable cytokines: A matched pair analysis. J. Inflamm. Res..

[14] Fischer C., Doll J., Tanner M., Bruckner T., Zimmermann G., Helbig L., Biglari B., Schmidmaier G., Moghaddam A. (2016). Quantification of tgf-ss1, pdgf and igf-1 cytokine expression after fracture treatment vs. Non-union therapy via masquelet. Injury.

[15] Haubruck P., Heller R., Apitz P., Kammerer A., Alamouti A., Daniel V., Schmidmaier G., Moghaddam A. (2018). Evaluation of matrix metalloproteases as early biomarkers for bone regeneration during the applied masquelet therapy for non-unions. Injury.

[16] Heller R.A., Raven T.F., Swing T., Kunzmann K., Daniel V., Haubruck P., Akbar M., Grutzner P.A., Schmidmaier G., Biglari B. (2017). Ccl-2 as a possible early marker for remission after traumatic spinal cord injury. Spinal Cord.

[17] Masquelet A.C., Obert L. (2010). Induced membrane technique for bone defects in the hand and wrist. Chirurgie de la Main.

[18] Bosemark P., Perdikouri C., Pelkonen M., Isaksson H., Tagil M. (2015). The masquelet induced membrane technique with bmp and a synthetic scaffold can heal a rat femoral critical size defect. J. Orthop. Res. Off. Publ. Orthop. Res. Soc..

[19] Piscaglia F., Nolsoe C., Dietrich C.F., Cosgrove D.O., Gilja O.H., Bachmann Nielsen M., Albrecht T., Barozzi L., Bertolotto M., Catalano O. (2012). The efsumb guidelines and recommendations on the clinical practice of contrast enhanced ultrasound (ceus): Update 2011 on non-hepatic applications. Ultraschall Med..

[20] Kuhlman J.E., Fishman E.K., Magid D., Scott W.W., Brooker A.F., Siegelman S.S. (1988). Fracture nonunion: Ct assessment with multiplanar reconstruction. Radiology.

[21] Savolaine E.R., Ebraheim N. (2000). Assessment of femoral neck nonunion with multiplanar computed tomography reconstruction. Orthopedics.

[22] Slade J.F., Gillon T. (2008). Retrospective review of 234 scaphoid fractures and nonunions treated with arthroscopy for union and complications. Scand. J. Surg..

[23] Akaike H., Lovric M. (2011). Akaike’s information criterion. International Encyclopedia of Statistical Science.

[24] Moghaddam A., Sperl A., Heller R., Kunzmann K., Graeser V., Akbar M., Gerner H.J., Biglari B. (2016). Elevated serum insulin-like growth factor 1 levels in patients with neurological remission after traumatic spinal cord injury. PLoS ONE.

[25] Moghaddam A., Heller R., Daniel V., Swing T., Akbar M., Gerner H.J., Biglari B. (2017). Exploratory study to suggest the possibility of mmp-8 and mmp-9 serum levels as early markers for remission after traumatic spinal cord injury. Spinal Cord.

[26] Moghaddam A., Sperl A., Heller R., Gerner H.J., Biglari B. (2016). Scd95l in serum after spinal cord injury. Spinal Cord.

[27] R Development Core Team (2015). R: A Language and Environment for Statistical Computing.

[28] Robin X., Turck N., Hainard A., Tiberti N., Lisacek F., Sanchez J.C., Muller M. (2011). Proc: An open-source package for r and s+ to analyze and compare roc curves. BMC Bioinform..

[29] Wickham H. (2009). Ggplot2: Elegant Graphics for Data Analysis.

[30] Bender D., Haubruck P., Boxriker S., Korff S., Schmidmaier G., Moghaddam A. (2015). Validity of subjective smoking status in orthopedic patients. Ther. Clin. Risk Manag..

[31] Calori G.M., Colombo M., Mazza E.L., Mazzola S., Malagoli E., Marelli N., Corradi A. (2014). Validation of the non-union scoring system in 300 long bone non-unions. Injury.

[32] Krammer D., Schmidmaier G., Weber M.A., Doll J., Rehnitz C., Fischer C. (2018). Contrast-enhanced ultrasound quantifies the perfusion within tibial non-unions and predicts the outcome of revision surgery. Ultrasound Med. Biol..

[33] Qi-hua X., Chen Z., Jian-gang Z., Da-zhong Z., Yong-qiang Z. (2014). Comparison of contrast-enhanced ultrasonography and contrast-enhanced mri for the assessment of vascularization of hydroxyapatite orbital implants. Clin. Imaging.

[34] Holstein J.H., Karabin-Kehl B., Scheuer C., Garcia P., Histing T., Meier C., Benninger E., Menger M.D., Pohlemann T. (2013). Endostatin inhibits callus remodeling during fracture healing in mice. J. Orthop. Res..

[35] Delany A.M., Hankenson K.D. (2009). Thrombospondin-2 and sparc/osteonectin are critical regulators of bone remodeling. J. Cell Commun. Signal..

[36] Taylor D.K., Meganck J.A., Terkhorn S., Rajani R., Naik A., O’Keefe R.J., Goldstein S.A., Hankenson K.D. (2009). Thrombospondin-2 influences the proportion of cartilage and bone during fracture healing. J. Bone Miner. Res..

[37] Yan M., Hu Y., Yao M., Bao S., Fang Y. (2018). Gm-csf ameliorates microvascular barrier integrity via pericytes-derived ang-1 in wound healing. Wound Repair Regen..

[38] Locksley R.M., Killeen N., Lenardo M.J. (2001). The tnf and tnf receptor superfamilies: Integrating mammalian biology. Cell.

